# P103: Contamination risk of alcohol-based hand disinfectants and skin antiseptics with bacterial spores

**DOI:** 10.1186/2047-2994-2-S1-P103

**Published:** 2013-06-20

**Authors:** K Steinhauer, B Meyer, C Ostermeyer, H-J Rödger, M Hintzpeter

**Affiliations:** 1 Product Development Hygiene / Bioscience, Schülke & Mayr GmbH, Norderstedt, Germany; 2 Ecolab Deutschland GmbH, Düsseldorf, Germany; 3 Bode Chemie GmbH, Hamburg, Germany; 4 Lysoform Dr. Hans Rosemann GmbH, Berlin, Germany; 5 B. Braun Melsungen AG, Melsungen, Germany

## Introduction

Alcohol-based products are regarded as most appropriate in hand disinfection and in skin antisepsis. While alcohols have an immediate microbicidal effect against all vegetative microbial forms, they do not readily kill bacterial spores. This fact raised the question of the contamination risk of alcohol-based hand disinfectants and skin antiseptics with bacterial spores.

## Objectives

The aim of this study was to evaluate the overall risk of hand disinfectants and skin antiseptics to get contaminated with bacterial spores throughout the production process. Additionally the risk of contamination with bacterial spores throughout the subsequent in-use period was investigated.

## Results

Besides raw materials, primary packaging was identified as a potential source of bacterial spores. Investigation of a total of 625 containers did not yield any microbial growth in 542 cases. Median colony count for aerobic spore-forming bacteria was 0.2 cfu/10 ml container content. No anaerobic spore-forming bacteria were detected.

Additionally, long-term survival of bacterial spores in aliphatic C2-C3 alcohols was investigated. 1-propanol was found to reduce the number of spores most effectively, with 2-propanol and ethanol having a somewhat less pronounced impact. Thus 1-propanol was found to give reduction rates of 1.35 lg after 7 weeks contact time at a concentration of 30% (v/v), and viability of *B. subtilis* spores was further decreased to > 1.5 lg by 30% (v/v) 1-propanol after 14 weeks.

Exemplary in-use tests of a typical hand disinfectant and a typical skin antiseptic did not detect any microbial contamination or change in the physico-chemical properties of the tested products over 12 months.

## Conclusion

Investigation of primary packaging material and in-use were found not to pose a hygienic risk for typical alcohol-based disinfectants regarding bacterial spores. Data from this study revealed that hygienic safety regarding contamination with bacterial spores can be achieved if appropriate production processes are in place. In order to control hygienic safety of alcohol-based hand rubs and antiseptics, a microbial limit of <1 cfu/10 ml is suggested as a quality-control threshold for finished goods.

## Disclosure of interest

K. Steinhauer Employee of Schülke & Mayr GmbH, B. Meyer Employee of Ecolab Deutschland GmbH, C. Ostermeyer Employee of Bode Chemie GmbH, H.-J. Rödger Employee of Lysoform Dr. Hans Rosemann GmbH, M. Hintzpeter Employee of B. Braun Melsungen AG

